# Identification and Expression Analysis of the *Solanum tuberosum StATG8* Family Associated with the WRKY Transcription Factor

**DOI:** 10.3390/plants11212858

**Published:** 2022-10-26

**Authors:** Injeong Song, Suji Hong, Sung Un Huh

**Affiliations:** Department of Biological Science, Kunsan National University, Gunsan 54150, Korea

**Keywords:** autophagy, autophagy-related (ATG) protein, ATG8, WRKY transcription factor, ATG8-interacting motif (AIM)

## Abstract

Autophagy is an evolutionarily well-conserved cellular catabolic pathway in eukaryotic cells and plays an important role in cellular processes. Autophagy is regulated by autophagy-associated (ATG) proteins. Among these ATG proteins, the ubiquitin-like protein ATG8/LC3 is essential for autophagosome formation and function. In this study, the potato *StATG8* family showed clade I and clade II with significantly different sequences. Expression of the *StATG8* family was also increased in senescence. Interestingly, the expression of the *StATG8* and other core *StATG* genes decreased in potato tubers as the tubers matured. The *StATG8* family also responded to a variety of stresses such as heat, wounding, salicylic acid, and salt stress. We also found that some Arabidopsis WRKY transcription factors interacted with the StATG8 protein *in planta*. Based on group II-a WRKY, StATG8-WRKY interaction is independent of the ATG8 interacting motif (AIM) or LC3 interacting region (LIR) motif. This study showed that the StATG8 family had diverse functions in tuber maturation and multiple stress responses in potatoes. Additionally, StATG8 may have an unrelated autophagy function in the nucleus with the WRKY transcription factor.

## 1. Introduction

Climate change has a significant impact on the productivity of various crops [1]. High temperatures, drought stress, floods, etc. have serious adverse effects on crop production. Since this adversely affects global food production, developing crops through novel molecular genetic engineering using various plant genes is required [2,3].

Selective macroautophagy/autophagy is one of the most evolutionarily conserved mechanisms and is a catabolic process induced primarily with nutrient deprivation and various stress conditions [4]. Autophagy is a basic function of eukaryotic cells and is well conserved in animals, plants, yeast, and fungi [5]. The most typical morphological feature of autophagy is the formation of bilayer autophagosomes containing selected cargo or non-selective bulk cytoplasm [4]. Autophagy is formed to remove dysfunctional organelles, abnormal cytoplasmic proteins, and pathogenic proteins; it eventually fuses with lysosomes (animals) or vacuoles (yeast and plants) to remove and recycle material [6]. Completion of the autophagosome forms a key step in autophagy that depends on a series of autophagy-related (ATG) proteins [7]. 

Among various ATG proteins, ATG8/LC3, a ubiquitin-like protein, performs almost all major processes of autophagosome formation [8]. Therefore, ATG8 protein is mainly used as an autophagosome marker to evaluate the induction of autophagy [9,10]. ATG8 is also required for cargo recognition through specific interactions with autophagy receptors during selective autophagy [11]. ATG8 is expressed as a cytoplasmic precursor that is processed at the C-terminal Gly residue by the ATG4 protein and is conjugated to phosphatidylethanolamine (PE) [12]. Lapidated ATG8 (ATG8-PE) protein is anchored to the inner and outer membranes of phagocytes [12]. The ATG8-PE protein serves as an adapter or scaffold for recruiting LC3 interacting region (LIR) motif-containing proteins in mammalian selective autophagy [13]. The term Atg8/ATG8 interaction motif (AIM) is also usually used in yeast and plants [14,15]. It was recently identified that the ubiquitin interaction motif (UIM) is a 20 amino acid stretch of amino acid sequence that folds into a single α-helix with a consensus sequence for ATG8 (φ-θ-X-A-φ-X-X-S) [16]. ATG8 binds to UIM and proteins via an alternative interaction site called the UIM docking site (UDS) [16]. The UIM-UDS interface greatly expands the scope of autophagy receptors and adapters in selective autophagy.

The *ATG* gene is evolutionarily conserved throughout eukaryotes [8]. However, the plant *ATG8* gene family is diversified in higher plants. Yeast and fungi have only one *ATG8* gene, but eleven *ATG8* isoforms have been identified in soybeans; seven in rice and potatoes, five in pepper and maize, and nine in Arabidopsis [17]. As expected, the *ATG8* gene family has been greatly expanded in plants to adapt to adverse and stressful conditions. The plant *ATG8* gene can be classified into two clades. Most members of the plant *ATG8* family are clade I and are associated with fungi, whereas clade II is more similar to animal *ATG8* homologues [18]. 

Recently, transcriptional regulators regulating *ATG* gene expression in animal cells, yeast, and plants have been identified [19,20]. For example, Arabidopsis TGA9 (TGA motif-binding protein 9) has been identified as a positive regulator of autophagy. Overexpression of *TGA9* enhanced *AtATG8b*, *AtATG8e*, and additional *ATG* genes via binding to the promoter [19]. In mammals, E2F transcriptional activity is regulated by several mechanisms, including autophagy genes. E2F1 upregulates the expression of four autophagy genes, *microtubule-associated protein-1 light chain-3* (*LC3*), *ATG1*, *ATG5*, and the *damage-regulating autophagy regulator* (*DRAM*) through binding of the *ATG* promoter [21,22]. Therefore, it is quite natural that the expression of the autophagy gene is regulated by transcriptional regulators. However, in the case of plants, it can be expected that the expression control function will be diversified due to the expansion of the *ATG8* gene. Additionally, mammalian LC3B and plant ATG8 are localized both in the cytoplasm and the nucleus [23,24,25]. Several studies have shown that chromatin and nuclear membranes are targets of nuclear autophagy [23]. In fungi, *Fusarium graminearum* Atg8 can relocalize between the cytoplasm and the nucleus in an acetylation-dependent manner [26].

In this study, we investigated the *StATG8* family present in potatoes. The *StATG8* family showed specific response patterns at different stages of development and to different stresses. Additionally, StATG8, corresponding to clade I, interacted with the WRKY transcription factor; WRKY may affect the expression of *StATG8*. Although not yet known, additional functions of StATG8 in the nucleus are expected to be related to transcriptional regulators.

## 2. Results

### 2.1. The Potato StATG8 Family Contained Two Distinct Subgroups

**Figure 1:**
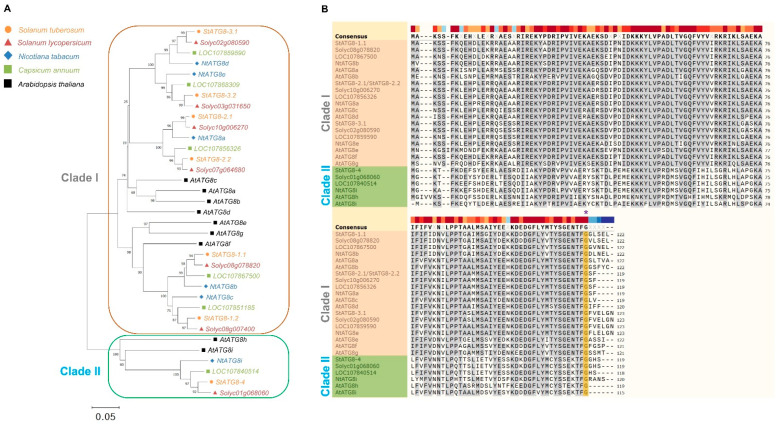
Potato *StATG8* family divided into two group with other plant species. (**A**). Phylogenetic relationships of *ATG8s* from *Solanum tuberosum* with those of *Nicotiana tabacum*, *Solanum lycopersicum*, *Capsicum annuum*, and *Arabidopsis thaliana*. The tree was constructed from an analysis conducted with MEGA-X software using a maximum likelihood method. Phylogenetic tree was based on the nucleotide sequence data. Numbers along the branches (bootstrap value) show the percentage occurrence of nodes in 1000 replicates of stimulation. Sequences of *ATG8* genes: *StATG8-1.1*; *XM_006355410.2*, *StATG8-1.2*; *XM_006358290.2*, *StATG8-2.1*; *XM_006352449.2*, *StATG8-2.2*; *XM_006346145.2*, *StATG8-3.1*; *XM_006348060.2*, *StATG8-3.2*; *XM_006343223.2*, *StATG8-4*; *Soltu.DM.01G025400.1*, *Solyc08g078820*; *XM_010326987.3*, *Solyc08g007400*; *XM_004244522.4*, *Solyc07g064680*; *NM_001247701.2*, *Solyc10g006270*; *XM_004248315.4*, *Solyc02g080590*; *XM_010318289.3*, *Solyc03g031650*; *XM_004234427.4*, *Solyc01g068060*; *NM_001247710.1*, *NtATG8a*; *KR336564.1*, *NtATG8b*; *KR336565.1*, *NtATG8c*; *KR336566.1*, *NtATG8d*; *KR336567.1*, *NtATG8e*; *KR336568.1*, *NtATG8i*; *XM_016590903.1, AtATG8a*; *NM_118319.4*, *AtATG8b*; *NM_001340494.1*, *AtATG8c*; *NM_104884.5*, *AtATG8d*; *NM_126586.4*, *AtATG8e*; *NM_130080.6*, *AtATG8f*; *NM_117751.4*, *AtATG8g*; *NM_115928.5*, *AtATG8h*; *NM_111517.3*, *AtATG8i*; *NM_112426.4*, *LOC107840514*; *XM_047398402.1*, *LOC107851185*; *XM_047402075.1*, *LOC107867500*; *XM_016713764.2*, *LOC107856326*; *XM_016701325.2*, *LOC107868309*; *XM_016714970.2*, and *LOC107859590*; *XM_016704642.2* were retrieved from the GenBank database (https://www.ncbi.nlm.nih.gov/genbank/) on 1 to 10 January 2022. (**B**). Alignment of multiple amino acid sequences of the StATG8 proteins with other plants using the neighbor-joining. Yellow box indicates the conserved glycine essential for lipidation.

A genome-wide analysis of the *ATG* family present in various crops is ongoing by many researchers [27,28,29,30,31]. Unlike other eukaryotes, the plant *ATG8* family has been shown to diversify the gene family. Therefore, research on the *ATG8* family present in many plants is ongoing. In the case of potatoes (*Solanum tuberosum* L.), only *in-silco* data were analyzed and there were few actual experimental results [32]. Therefore, we identified the *StATG8* family (Table S1). Additionally, we analyzed the DNA and protein sequences of the potato *ATG8* family. The difference was confirmed by analyzing the DNA sequence of the *ATG8* family present in red pepper, tobacco, and tomato, which are representative crops of the Solanaceae family together with potato. *ATG8* is divided into two main types as previously reported according to DNA sequencing [17,32]. The difference was so large that it was confirmed that there was only one clade II in each crop of Solanaceae (Figure 1A). In addition, we found that one clade II gene was also present in pepper (Figure 1A). The results of the comparative analysis of protein sequences were even more interesting. There is a significant difference between the two clades (Figure 1B). The StATG8-2.1 and StATG8-2.2 protein sequences are identical, but the DNA is slightly different (Figure 1A,B). There may be specific functional differences between the two genes due to differences in promoters.

Mature ATG8, processed by the ATG4 protease, is conjugated to the membrane lipid phosphatidylethanolamine (PE) and functions as a ubiquitin-like protein required for autophagosome formation [33]. The cysteine proteinase ATG4 has a dual function in autophagosome assembly and degradation by exposing the C-terminal glycine residue of ATG8 [34]. Interestingly, members of the clade II group of Arabidopsis AtATG8h and AtATG8i were already glycine-exposed (Figure 1B). However, StATG8-4, NtATG8i, CaLOC107840514, and Solyc01g068060 may require ATG4 cleavage for conjugation and deconjugation in autophagosome formation (Figure 1B). These evolutionary consequences of ATG8 clades I and II with large differences in amino acid sequence are probably characteristic of Solanaceae crops.

### 2.2. Promoters of StATG8s Contained Multiple Regulatory Cis-Elements

**Figure 2:**
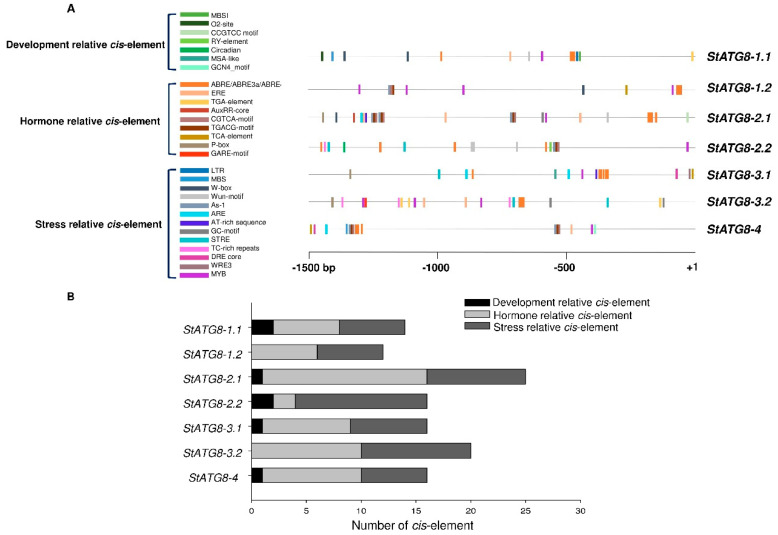
Promoter analysis of *StATG8* family. (**A**). The 1.5 kb genomic DNA sequence upstream of the initiation codon of each *StATG* was retrieved from the *S*. *tuberosum* genome (http://spuddb.uga.edu/index.shtml) on 1 to 20 February 2022. The *cis*-elements of *StATG8* promoter were predicted using PlantCARE (http://bioinformatics.psb.ugent.be/webtools/plantcare/html/) on 1 to 20 February 2022. (**B**). The *cis*-element present in *StATG8* is divided into three categories related to development, hormone, and stress. It was summarized in a graph.

The regulatory elements of the *StATG8* promoter sequence were analyzed to understand regulatory pathways and gene functions. The 1.5 kb protomer sequence of *StATG8* was selected through the Spud Potato Genomics Resource DB (http://spuddb.uga.edu/) on 1 to 20 February 2022. Specific *cis*-acting regulatory elements of the *StATG8* gene were identified using the PlantCARE database (http://bioinformatics.psb.ugent.be/webtools/plantcare/html/ on 1 to 20 February 2022) [35]. As shown in Figure 2 and Table 1, at least 15 different *cis*-elements were identified in the *StATG8* promoter involved in development, hormone, and stress responsiveness.

In the promoters of *StATG8-2.1* and *StATG8-2.2* having the same protein sequence, the proportion of hormone-related *cis*-elements in the promoters was relatively higher in *StATG8-2.1* than in *StATG8-2.2* (Figure 2B). The promoter of *StATG8-2.1* has about 35% more regulatory elements than other *StATG8* members. Except for the *StATG8-2.2* promoter, the abscisic acid (ABA)-responsive element ABRE was present in all *StATG8* promoters [36]. Moreover, MYB *cis*-elements are found in all *StATG8* promoters, which may be associated with drought stress [37]. Regulatory elements corresponding to methyl-jasmonic acid (MeJA)-responsiveness such as CGTCA/TGACG-motifs were also found [38]. Many of the *StATG8* promoters exhibited wounding-response elements such as As-1, Wun, and WRE3 motifs [39,40]. It can be expected that the expression of *StATG8* may also be affected by mechanical stress and immune responses [41]. WRKY transcription factor binding motif “W-box” was also found in the promoters of *StATG8-1.1*, *StATG8-1.2*, and *StATG8-2.1*. It can be expected that *StATG8* genes can be regulated using the WRKY transcription factor [42]. From the previous reports, autophagy plays an important role in development [43,44]; the regulatory elements CCGTCC motif, RY-element, MSA-like, and GCN4-motif related to development were present in *StATG8* promoters (Figure 2 and Table 1) [45]. Thus, the diverse *StATG8* genes are deeply associated with multiple stress responses and developments. 

### 2.3. The Potato ATG8 Family Genes Are Specifically Regulated during Leaf Senescence and Tuber Maturation 

**Figure 3:**
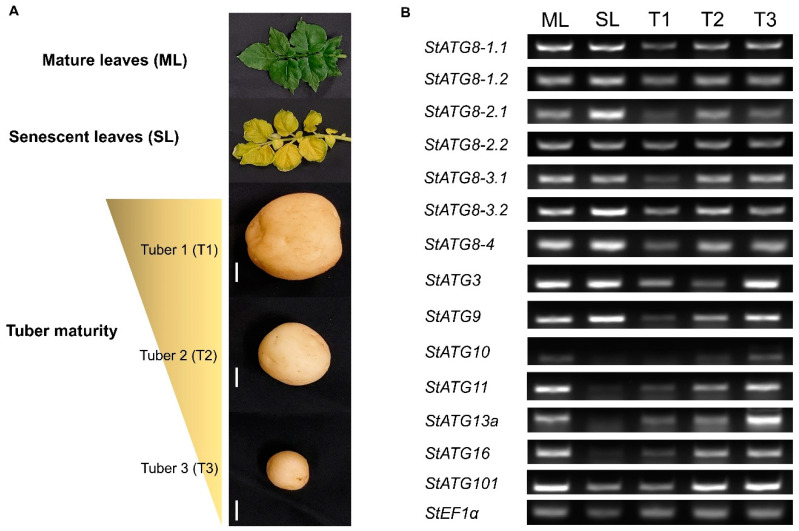
Expression patterns of *StATGs* in mature leaves, senescent leaves, and tubers. (**A**). Photographs of fully mature leaves, senescent leaves, and tubers of potato plants were used for sampling. (**B)**. Semiquantitative RT-PCR measurement of *StATGs* expression in mature leaves (ML), senescent leaves (SL), and tubers (T1 to T3). *StEF1α* was used as a reference gene. The white bar represents 1 cm. Semi-quantitative RT-PCR was performed using the *StATG* gene-specific primers and primer information is in Supporting Table S2.

It is well known that the function of autophagy is directly related to cellular senescence [46,47]. In this regard, we investigated how the gene expression of the *StATG8* family changes during leaf senescence and tuber maturation, which is important for its value as a crop. In semi-quantitative RT-PCR comparing mature and senescent leaves (Figure 3A), *StATG8-2.1*, *StATG8-3.2*, and *StATG8-4* showed strong expression. On the other hand, *StATG8-1.1*, *StATG8-1.2*, *StATG8-2.2*, and *StATG8-3.1* did not exhibit a significant increase in gene expression (Figure 2B). Thus, the functions of *StATG8* in the aging process are not all the same. We also confirmed the expression of *StATG8* by sampling tubers grown in three different sizes (Figure 3A). Interestingly, it was observed that the expression of most *StATG8* genes decreased as tuber size increased (Figure 3B). It seems different from the pattern of increasing *ATG* genes during the fruit ripening of tomatoes [48]. We further investigated the expression levels of other core *StATG* genes present in potato. It was confirmed that the expression of *StATG10*, *StATG11*, *StATG13a*, *StATG16*, and *StATG101* was strongly down-regulated in leaf senescence (Figure 3B). In addition, it was confirmed that *StATG3*, *StATG9*, *StATG10*, *StATG11*, *StATG13a*, *StATG16*, and *StATG101* dramatically decreased according to tuber maturation as in the *StATG8* family genes (Figure 3B). It was first discovered that the expression of *StATG8* and core *StATG* genes decreases in potato tubers maturation. Based on the above results, we speculated that the autophagy could be important for tuber developments.

### 2.4. Response of StATG8 Genes Expression upon Multiple Stresses

**Figure 4:**
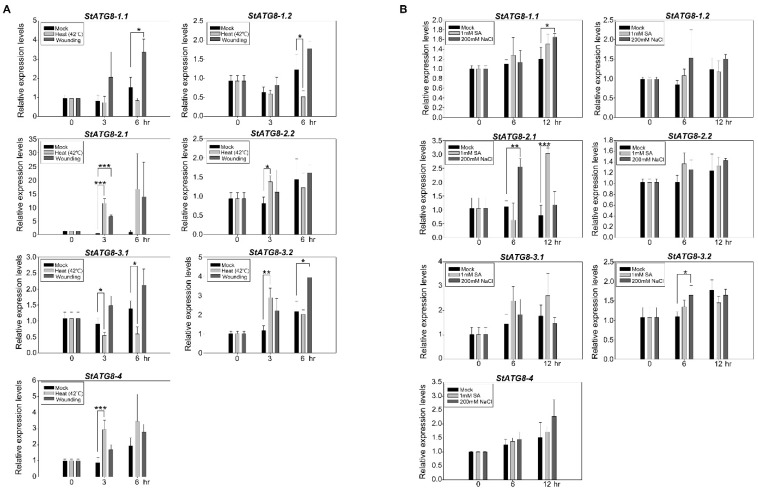
Expression analysis of *StATG8s* in response to multiple stresses. (**A**,**B**). Real-time PCR analysis of *StATG8* genes in response to heat (42 ℃), mechanical wounding (**A**), 1 mM salicylic acid (SA), and 200 mM NaCl treatments (**B**). For stress treatments, 4-week-old potato plants were used to stress treatment for the indicated times. *StEF1α* was used as a reference gene. Error bars indicate the standard deviations of three independent qRT-PCR biological replicates. Asterisks indicate significant differences from the control using the unpaired Student’s *t*-test (* *p* < 0.05, ** *p* < 0.01, and *** *p* < 0.001).

Through previous promoter analysis, we hypothesized that the *StATG8* family gene expressions would respond to wounding and heat stresses. Under heat stress, the expression of *StATG8* family genes was observed in two patterns. *StATG8-2.1*, *StATG8-2.2*, *StATG8-3.2*, and *StATG8-4* showed a strong increase in thermal stress at 3 h (Figure 4A). On the other hand, the expression of *StATG8-3.1* was significantly decreased, and *StATG8-1.1* and *StATG8-1.2* showed a tendency to decrease after 6 h in the heat stress (Figure 4A). Upon wounding stress, *StATG8-1.1*, *StATG8-2.1*, and *StATG8-3.2* showed an increased pattern (Figure 4A). These results suggest that the *StATG8* family will function in response to mechanical stress and wounding stress induced by insects.

We also investigated whether the *StATG8* family responds to the plant defense hormone salicylic acid (SA) [49]. Interestingly, only *StATG8-2.1* responded to SA at 12 h (Figure 4B). However, other *StATG8* genes did not change significantly to SA treatments between 6 and 12 h. The absence of a response by SA probably means that most *StATG8* genes can be regulated by other hormones such as JA and ABA. Additionally, salt stress is also one of the very important plant stresses and we tested whether *StATG8* family responses to salt stress. The gene expression of *StATG8-1.1*, *StATG8-2.1*, and *StATG3-2.2* showed a significantly upregulated pattern (Figure 4B). As a result, each member of *StATG8* specifically responds in a specific stress and its functions may be diversified.

### 2.5. Response of Core StATG Genes upon Multiple Stresses

**Figure 5:**
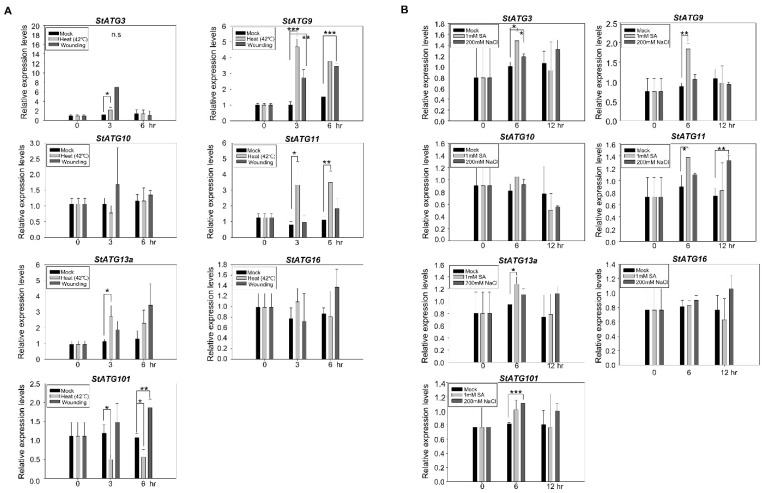
Core *StATG* genes responded to multiple stresses. (**A**,**B**). Real-time PCR analysis of core *StATG* genes in response to heat (42 ℃), mechanical wounding (**A**), 1 mM salicylic acid (SA), and 200 mM NaCl treatments (**B**). For stress treatments, 4-week-old potato plants were used for stress treatment for the indicated times. *StEF1α* was used as a reference gene. Error bars indicate the standard deviations of three independent qRT-PCR biological replicates. Error bars represent SD of three independent experiments. Asterisks indicate significant differences from the control using the unpaired Student’s *t*-test (* *p* < 0.05, ** *p* < 0.01, and *** *p* < 0.001).

Based on the results of investigating the gene expression of the *StATG8* family for various stresses, qRT-PCR was performed to test the expression pattern of the core *StATG* genes. The results exhibited that *StATG3*, *StATG9*, *StATG11*, *StATG13a*, and *StATG101* significantly responded to heat and wounding stresses (Figure 5A). On the other hand, mRNA transcript levels of *StATG10* and *StATG16* were not significantly changed upon heat and wounding stresses (Figure 5A). *StATG11* showed a specific increase pattern in heat stress. Transcript levels of *StATG9* were observed at increased patterns in both heat and wounding stresses. *StATG101* exhibited a down-regulated pattern in heat, whereas increased expression was observed at 6 h for wounding stress (Figure 5A). Like the *StATG8* family, the core *StATG* genes are also thought to perform autophagy function in response to heat and wounding stresses. 

We also investigated the expression patterns of core *StATG* genes during SA and NaCl treatments. The expression of *StATG3*, *StATG9*, *StATG11*, and *StATG13a* genes increased upon SA treatment (Figure 5B). Upon salt treatment, *StATG11* and *StATG101* showed a significant increase pattern, though the rest of the *StATG8* genes did not show a significant change of mRNA transcript levels (Figure 5B). Thus, several *StATG* genes responded to SA and salt treatments. These different types of responses are expected to have features that contribute to autophagy formation and function in multiple stresses.

### 2.6. StATG8 Interacts with WRKY Transcription Factor in Planta

The function of autophagy is based on what happens in the cytoplasm. However, it has recently been reported that animal LC3 and fungal Atg8, which corresponds to plant ATG8, exist in the nucleus and shuttle between the nucleus and the cytoplasm through post-translational modifications such as acetylation [23,26]. Furthermore, there are reports that cassava (*Manihot esculenta*) MeWRKY20 associated with MeATG8s and activated *MeATG8* gene expression in the nucleus [50]. In addition, transcriptional regulator TGA9 also regulate the expression of *AtATG8* genes [19]. Although the function of ATG8 in the nucleus is still largely unknown, we performed protein interaction experiments to determine whether StATG8 interacts with WRKY transcriptional regulators.

**Figure 6:**
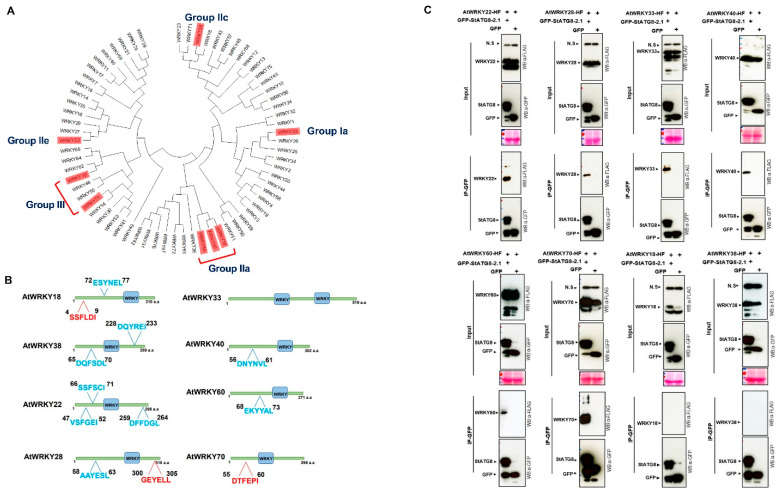
StATG8 associated with WRKY transcription factors *in planta*. (**A**) Phylogenetic relationships of protein sequences within the AtWRKY family. The tree was constructed from an analysis conducted with MEGA-X software using a maximum likelihood method [51]. AtWRKYs used for CoIP are shown in red boxes. (**B**) Analysis of putative ATG8 binding motifs present in AtWRKY utilizing the iLIR database on 1 to 25 June 2022 [52]. ATG8 interacting proteins have a LC3-interacting region (LIR) and Atg8-interacting motif (AIM) responsible for interaction with ATG8 family proteins. In AtWRKYs, LIR motifs are shown in red text and AIM motifs are shown in blue text. AtWRKY18, AtWRKY40, and AtWRKY60 protein sequence analysis corresponds to group II-a. Only AtWRKY18 contains a putative LIR motif at the N-terminal end. AtWRKY33 do not contain any ATG8-binding related motifs. (**C**) Co-immunoprecipitation (coIP) assays reveal that StATG8 is associated with multiple AtWRKY transcription factors. *Agrobacterium*-mediated transient co-expression of *GFP-StATG8-2.1* or *GFP* control with *AtWRKY-22-HF*, *AtWRKY-28-HF*, *AtWRKY-33-HF*, *AtWRKY-40-HF*, *AtWRKY-60-HF*, *AtWRKY-18-HF*, *AtWRKY-20-HF*, and *AtWRKY-38-HF*, and *AtWRKY-70-HF* was performed in *N. benthamiana* leaves. Anti-GFP co-IPs were performed with total protein extracts and probed with anti-GFP and -FLAG antibodies. All experiments were performed for at least three times.

We first selected several groups of Arabidopsis WRKY (Figure 6A). As shown in Figure 6B, we determined whether these proteins had AIM or LIR motifs known to be capable of interacting with ATG8 proteins using iLIR database (https://ilir.warwick.ac.uk on 1 to 20 February 2022) and hfAIM (http://bioinformatics.psb.ugent.be/hfAIM/ on 1 to 20 February 2022). Interestingly, most of the AtWRKY proteins contained one or two AIMs. In case of AtWRKY33, it does not have any AIM signatures and it could be used as a negative control in StATG8-WRKY interaction coIP experiments. 

We prepared co-immunoprecipitation (co-IP) samples with *35S::GFP-StATG8-2.1* and various *35S::AtWRKYs-HF* combinations for *Agrobacterium*-mediated transient assay using *Nicotiana benthamiana* [53,54]. As previously reported [50], we expected that there would be enough protein interaction between WRKY and StATG8. Surprisingly, AtWRKY33, which does not contain either AIM or iLIR (Figure 6B), can still associate with GFP-StATG8-2.1 in coIP (Figure 6C). However, AtWRKY18 containing both AIM and iLIR did not work in coIP with StATG8-2.1 (Figure 6B,C). AtWRKY38 has two AIMs (Figure 6B), but when co-expressing AtWRKY38 and StATG8-2.1, protein interactions could not be confirmed in coIP experiments (Figure 6C). In our coIPs, StATG8-2.1 can associate with many of AtWRKY proteins. However, additional experiments are needed to determine whether the expected AIM motif can play a major role.

## 3. Discussion

Autophagy is well conserved in all eukaryotes and is known to play a variety of roles in many areas, including intracellular immunity, development, stress response, and waste recycling [4,55,56]. In this study, we characterized the *StATG8* family among important *ATG* genes present in potatoes, a representative food resource under developmental and various stress conditions. Although the function of ATG8 in the nucleus is not yet clear, we found that StATG8 can interact with WRKY family proteins, one of the transcription factors, either using AIM or an unknown motif.

Plants are constantly exposed to various stresses [57]. Climate change, which has been emphasized, is creating a situation that disturbs the homeostasis of plants [58]. This climate condition is believed to be closely related to the function of autophagy, which plays an important role in maintaining intracellular homeostasis. To overcome this climate change, genetic engineering of autophagy can be one of the strategies to improve enhanced crop development. Research on core autophagy genes is ongoing [59,60]. One of them, ATG8 protein, plays an important role in the conjugation part in autophagosome formation. Plant ATG8 has several unique characteristics compared to other eukaryotes. First, plant ATG8 contains more isoforms than other eukaryotes. Animals with functions corresponding to plant *ATG8* (*LC3A-C*, *GABARAPs*, and *GATE-16*) and fungi have 1-2 isoforms of the gene [61,62], whereas Solanaceous crops such as potatoes have about 7-8 genes (Figure 1A). Furthermore, *StATG8* members are divided into two clades (Figure 1A). This suggests that the function of ATG8 is diversified as a plant-specific autophagy. In this study, we also confirmed that different features exist in each function in the gene expression and promoter analysis of *StATG8* family. Second, ATG8 is an effector target of foreign pathogens in plant immunity. For example, StATG8 is targeted by PexRD54, an effector of the Irish potato famine pathogen *Phytophthora infestans* [63]. PexRD54 induces ATG8 malfunction and perturbs plant autophagy-related immunity. Barley stripe mosaic virus γb protein also utilizes a strategy to increase viral infection by disrupting the ATG7-ATG8 Interaction [64]. The pathogen probably targets ATG8 to suppress the immune function of autophagy. Interestingly, there are very large changes in the protein sequence of StATG8 members [32]. StATG8 is divided into two major clades, which are probably related to the evolution of function.

Plant leaf senescence is one of the degenerative processes of organelles or biomolecules [65]. Studies of the function of senescence-related autophagy have fairly similar results in both animals and plants [66]. Autophagy is one of the functions contributing to the maintenance of basic homeostasis in plants and is thought to play a distinct role in the breakdown of recyclable substances during leaf senescence [4]. In our experimental results, it was confirmed that the expression of *StATG8-2.1*, *StATG8-3.2*, and *StATG8-4* was highly upregulated in leaf senescence (Figure 2B). In addition, it was found that not all core *ATG* genes increase mRNA levels during leaf senescence. That is, each isoform of the StATG8 family may have a different part of function in senescence-associated autophagosome formation. 

Potato tubers are one of the tissues used as a very important food resource [67]. To date, no information is known on the expression of autophagy-related genes in tuber formation. Through this study, we newly discovered that expression of the *StATG8* family decreased during tuber maturation. This is expected to be a part that can be used for engineering by regulating the function of autophagy that regulates tuber formation. Moreover, since core *ATG* genes also show a decrease (Figure 2B), autophagy activity is expected to decrease during tuber maturity. It is necessary to proceed with the study of tuber formation using Clustered Regularly Interspaced Short Palindromic Repeats and associated protein 9 (CRISPR-Cas9)-based genome editing and potato *StATG* gene overexpression system. In general, autophagy activity increases as the fruit ripens. For example, in the fruit maturation of strawberry, the protein expression of *ATG8* is increased and the ratio of ATG8-PE is increased from green fruits to red fruits [68]. Additionally, the level of NBR1 protein, a representative cargo protein, was also increased [68]. In the Solanaceae pepper, CaATG8 and CaATG5 were found to increase both protein and mRNA levels during the ripening of pepper fruit [69]. This result may be because the tuber of the potato is not a fruit, or it may be due to a difference in tissue composition.

Plants are exposed to and respond to multiple stresses [25,70]. Various *cis*-elements in the potato *StATG8* family were found to respond to MeJA, wounds, drought stress, etc. in promoter analysis (Figure 2). In fact, the *StATG8* member showed various expression patterns from the wound and SA treatment results related to biotic stresses. In particular, *StATG8-1.1*, *StATG8-2.1*, and *StATG8-3.2* showed significant and strong gene expression patterns in the wounding stress (Figure 4A). On the other hand, in the SA treatment, only *StATG8-2.1* exhibited significantly enhanced gene expression. Although previous studies have reported that ATG8 has multiple roles in immune processes [57], most StATG8s appear to have a specialized function in JA-related wounding stress. However, StATG8-2.1 maybe act in SA pathway in defense response. It is also worth investigating the response of *StATG8* genes to additional plant hormones.

Global warming associated with climate change adversely affects plant growth at high temperatures and severely limits crop productivity [71]. Autophagy plays an important role in plant heat tolerance, in part by breaking down unusable proteins caused by heat stress [72]. We found that the *StATG8* genes responded quite strongly to heat stress (Figure 4A). All gene expression patterns, except for *StATG8-1.1* and *StATG8-1.2*, were confirmed to be significant. This showed a similarly increased pattern for other core *ATG* genes. For example, there is a result that heat shock protein HsfA1a directly activates gene expression by binding to the heat-shock *cis*-element of Arabidopsis *ATG10* and *ATG18f* [73]. The autophagy gene is directly regulated by transcriptional regulators such as heat shock transcription factors and is likely regulated by temperature changes; certain members of the *StATG8* family are also expected to be involved.

Plant autophagy research is focused on the function in the cytoplasm where selective autophagy works [11]. However, recent studies have shown that ATG8 interacts with various proteins in the nucleus [74,75]. Pathogen proteins might affect autophagy activity through ATG8 interaction in the nucleus. The function of plant ATG8 in the nucleus is not yet well understood, but there may be interactions similar to those of almost all plants, such as the example of the protein interaction of MeATG8-MeWRKY20 [50]. In the disease resistance response, MeATG8 interacts with MeWRKY20 to increase autophagy activity and further enhances *ATG8* gene expression. Using the Arabidopsis AtWRKY22 homologue of MeWRKY20, we also confirmed protein interactions with StATG8 (Figure 6). This would be different from the general autophagy function of ATG8 in the nucleus. In addition, not all AtWRKYs interact with StATG8, which may be a versatile strategy to promote the autophagic activity of plant ATG8 with various ATG8s to respond to multiple stresses. 

Recently, it has been found that UIM as well as AIM exist [16]. It can be expected that the binding motifs used by ATG8 to interact with specific targets may not yet be limited. In our experiments, AtWRKY33, a group Ia member, interacted with StATG8 despite the absence of a motif that interacts with ATG8. AtWRKY18, AtWRKY40, and AtWRKY60 formed a small group IIa with AIM but only AtWRKY18 exhibited no association with StATG8. It would be a good comparison if further studies on protein–protein interactions and *StATG8* gene expression activation were performed using the potato-WRKY homologue group. Therefore, it is possible that unknown ATG8-binding motifs exist. Further studies are needed to determine whether the interaction of ATG8-WRKY is one of the regulatory actions that play a positive role in autophagy activity or autophagosome formation. If this regulatory action is common, it can be applied for crop development through dual regulation of ATG8 and WRKY. 

**Figure 7:**
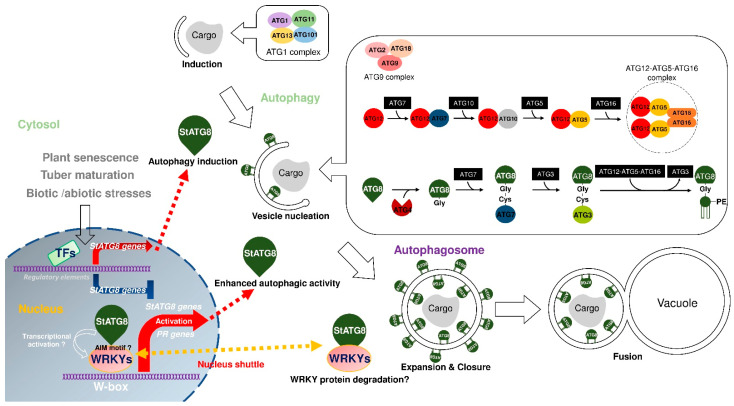
A model for the functional diversification of potato StATG8 under multiple stress conditions. The model for autophagy formation and *ATG8* gene activation was inspired by following papers [50,56,76,77].

Although functional studies of the StATG8 family are not yet complete, we presented a working model of the potato StATG8 family (Figure 7). The expression of seven members of *StATG8* is regulated by specific transcription factors during leaf senescence and tuber maturation. The expression of *StATG* genes decreases with increasing tuber maturation. Additionally, specific *StATG8* genes are up-regulated upon heat, saline, wounding, and salicylic acid treatments, ultimately contributing to autophagy formation and overcoming the stresses. The WRKY transcription regulator is expected to play a role in regulating the gene expression of *StATG8*. Although the exact mechanism is not yet known, StATG8 proteins can associate with WRKY proteins through specific AIM or unknown binding motifs. It is expected to enhance the expression of *StATG8* and activate autophagy activity. On the other hand, a negative feedback-regulation can be expected in which StATG8 pulls WRKY out of the nucleus and degrades it.

## 4. Materials and Methods

### 4.1. Plant Materials and Stress Treatments

*Arabidopsis thaliana* (Col-0), tobacco (*Nicotiana benthamiana*), and potato (*Solanum tuberosum* L. cv. Sumi) plants were potted in soil and placed in a growth chamber under controlled conditions with 60% relative humidity and a 16 h:8 h, light:dark, 22 °C condition. Four-week-old potato plants were used for salt, wounding, heat, and SA hormone treatments at the indicated times.

### 4.2. Identification of StATG8 Genes in Solanum tuberosum

To identify the potato *StATG8s*, protein and coding sequences searched the potato genome databases (https://solgenomics.net/ on 1 to 20 February 2022) and Spud DB database (http://solanaceae.plantbiology.msu.edu/ on 1 to 20 February 2022) with default parameters. Physiochemical properties of StATG8s protein length, molecular weight, and Isoelectric point (pI) were calculated using ExPASy Prot-Param tool (http://web.expasy.org/protparam/ on 1 to 20 February 2022) [78]. SMART (http://smart.embl-heidelberg.de/ on 1 to 20 February 2022) was used to identify the conserved domains [79].

### 4.3. Phylogenetic Analysis and Protein Sequence Alignment

Phylogenetic analysis was performed for potato *StATG8* with *Nicotiana tabacum* (Nt), *Solanum lycopersicum* (Sl), *Capsicum annuum* (Ca), and *Arabidopsis thaliana* (At). The tree was constructed from an analysis conducted with MEGA-X software using a maximum likelihood method [51]. Phylogenetic tree was based on the nucleotide sequence data. Numbers along the branches (bootstrap value) show the percentage occurrence of nodes in 1000 replicates of stimulation. Solanaceae crops and the identified StATG8 proteins were compared using ClustalW with basic parameters. 

### 4.4. Cis-Acting Element Analysis in StATG8 Promoter Regions

1.5 kb upstream region from 5′ genomic DNA of *StATG8* was obtained from the Spud database (http://potato.plantbiology.msu.edu/ on 1 to 20 February 2022). The promoter sequences were analyzed using PlantCARE (http://bioinformatics.psb.ugent.be/webtools/plantcare/html/ on 1 to 20 February 2022) database [35]. 

### 4.5. RNA Extraction, Quantitative Real-Time PCR, and Semi-Quantitative RT-PCR

RNA isolation was performed using RNAiso Plus (Takara Bio, Shiga, Japan) according to the instructions provided. The RNA samples were quantified and 0.5 μg of RNA was taken for cDNA synthesis using M-MLV Reverse Transcriptase cDNA synthesis kit (ELPIS BIOTECH, Daejeon, Republic of Korea) as per manufacturer's instructions. Quantitative RT-PCR was performed using specific primers to *StATG8* genes using SYBR green kit (Kapa Biosystems, Wilmington, USA). The conditions for qRT-PCR were as follows: 1 min at 95 °C, followed by 35 cycles of 30 s at 95 °C, 30 s at 55-58 °C, and 30 s at 72 °C. The relative expressions of *StATG8* were calculated by normalizing the PCR threshold cycle (Ct) values to the expression of reference genes *StActin* or *StEF1a* [80]. Semi-quantitative RT-PCR was performed as follows: 1 min at 95 °C, followed by 27 cycles of 30 s at 95 °C, 30 s at 55–58 °C, and 30 s at 72 °C. The primers are listed in the supporting Table S2.

### 4.6. Plasmid Constructs

*35S::GFP-StATG8s* were cloned previously using Gateway cloning into the destination vector PK7WGF2 (N-terminal GFP) [63]. *35S::AtWRKYs-HF*(His-Flag) were cloned previously using GoldenGate cloning into the pICH86988 (C-terminal 6×His/3×FLAG tag) [81].

### 4.7. Co-Immunoprecipitation

*A. tumefaciens* GV3101 strains carrying the plant expression constructs were diluted in an agroinfiltration medium (10 mM MgCl_2_, 5 mM 2-[Nmorpholine]-ethanesulfonic acid [MES], pH 5.6) to a final OD_600_ of 0.5. For transient co-expression assays, agro-cells were infiltrated in *N. benthamiana* [53]. The leaves were ground into a fine powder in liquid nitrogen with a mortar and pestle. Ground tissue was mixed with a GTEN buffer (150 mM Tris-HCl, pH 7.5; 150 mM NaCl; 10% (*w*/*v*) glycerol; 10 mM EDTA) augmented with 10 mM dithiothreitol, 2% (*w*/*v*) PVPP, and 1% (*v*/*v*) protease inhibitor cocktail (Sigma, MO, USA). Co-IP was performed following the protocol described previously [54]. Immunoprecipitation was performed using GFP Trap A beads (Proteintech, Manchester, UK) and mixing the beads by turning end-over-end for two hours in the cool room. Immunoblot was performed with anti-GFP (Proteintech, Manchester, UK) and anti-FLAG (Sigma, MO, USA) antibodies.

## 5. Conclusions

Although the plant ATG8 family exhibits great redundancy, we found that potato *StATG8* gene members are subject to specific regulatory actions at multiple stresses or developmental stages. Furthermore, the protein interaction between StATG8 and WKRY transcription factor may be one of the mechanisms regulating autophagy. Further studies are needed to determine whether StATG8 works with WRKY to enhance *StATG8* gene expression or plays a role in supporting the proteolysis of WRKY protein.

## Figures and Tables

**Table 1:** *Cis*-acting regulatory element analysis in potato *StATG8* promoter regions.

	*Cis*-element	Functions	*StATG8-1.1*	*StATG8-1.2*	*StATG8-2.1*	*StATG8-2.2*	*StATG8-3.1*	*StATG8-3.2*	*StATG8-4*
**Development**	MBSI	MYB binding site involved in flavonoid biosynthetic genes regulation	1						
	O2-site	*Cis*-acting regulatory element involved in zein protein metabolism regulation	1						
	CCGTCC motif	involved in growth and development / meristem-specific regulatory			1				
	RY-element	*Cis*-acting regulatory element involved in seed-specific regulation				1			
	Circadian	*Cis*-acting regulatory element involved in circadian control				1			
	MSA-like	*Cis*-actin element involved in cell cycle regulation					1		
	GCN4_motif	*Cis*-regulatory element involved in endosperm expression							1
**Hormone**	ABRE/ABRE3a/ABRE4	*Cis*-acting element involved in the abscisic acid responsiveness	4	3	4		6	3	3
	ERE	Ethylene responsive element	1		3			2	1
	TGA-element	auxin-responsive element	1					3	
	AuxRR-core	*Cis*-acting regulatory element involved in the auxin responsiveness			1				
	CGTCA-motif	*Cis*-acting regulatory element involved in the MeJA -responsiveness		1	3	1			2
	TGACG-motif	*Cis*-acting regulatory element involved in the MeJA -responsiveness		1	3	1			2
	TCA-element	*Cis*-acting element involved in salicylic acid responsiveness		1			1		1
	P-box	gibberellin-responsive element			1		1	1	
	GARE-motif	gibberellin-responsive element						1	
**Stress**	LTR	*Cis*-acting element involved in low-temperature responsiveness	1						
	MBS	MYB binding site involved in drought-inducibility	1						1
	W-box	WRKY binding site involved in abiotic stress and defense response	2	1	1				
	Wun-motif	Wound-responsive element	1		1	3			
	As-1	drought and wound stress responsive elements		1	3	1			2
	ARE	*Cis*-acting regulatory element essential for the anaerobic induction			1	4	2		1
	AT-rich sequence	element for maximal elicitor-mediated activation			1		1		
	GC-motif	enhancer-like element involved in anoxic specific inducibility			1			2	
	STRE	Stress-responsive element				2	1	2	
	TC-rich repeats	*Cis*-acting element involved in defense and stress responsiveness				1		3	
	DRE core	Dehydration-responsive element					1		1
	WRE3	Wound and pathogen response					1		
	MYB	MYB binding site involved in drought-inducibility	1	4	1	2	1	3	1

## Data Availability

The data presented in this study are available on request from the authors. Informed consent was obtained from all subjects involved in the study.

## Supplementary Materials

The following supporting information can be downloaded at: https://www.mdpi.com/article/10.3390/plants11212858/s1, Table S1: StATG8 family identification; Table S2: RT-PCR/qRT-PCR primers.
