# Supplementary material for: Identification and Expression Analysis of the Solanum tuberosum StATG8 Family Associated with the WRKY Transcription Factor
Source: Plants (Basel). 2022 Oct 26;11(21):2858. doi: 10.3390/plants11212858 (PMC9659186; doi:10.3390/plants11212858)
Supplement: Supplementary file 1 [file plants-11-02858-s001.zip › plants-1981830-supplementary.pdf]

Supporting information (Tables S1-S2)

Table.S1 StATG8 family identification

gene name	sequence ID	chromosomal location	mRNA Genomic Coords(5'-3')	CDS length (nt)	protein length (aa)	Molecular weight (kD)	Isoelectric point (pI)
StATG8-1.1	Soltu.DM.08G025140	chr08	54968538 - 54972445	369	122	13.87	7.85
StATG8-1.2	Soltu.DM.08G000550	chr08	1066827 - 1063140	369	122	14.03	7.86
StATG8-2.1	Soltu.DM.10G001410	chr10	1164062 - 1161205	360	119	13.74	8.78
StATG8-2.2	Soltu.DM.07G026360.1 (XM_006346145.2)	chr07	55823203-55829137	360	119	13.74	8.78
StATG8-3.1	Soltu.DM.02G020190.3	chr02	34400493 - 34403324	372	123	14.05	8.75
StATG8-3.2	Soltu.DM.03G002690.2	chr03	2568108 - 2564884	372	123	14.15	5.89
StATG8-4	Soltu.DM.01G025400.1	chr01	64775470 - 64769358	360	119	13.66	6.73

Supporting information

Table. S2 RT-PCR/qRT-PCR primers

gene	F/R	Primer Sequence(5'-3')	gene	F/R	Primer Sequence(5'-3')
StEF1a	F	TTTGGCCCTACTGGTTTGAC	*StATG3*	F	ACAGACCCTGCTACCCTTCA
	R	GGGTCATCCTTGGAGTTTGA		R	GTTTCCCTGGAAGATGTGGA
StATG8-1.1	F	GACAATGTGCTACCGCCAAC	*StATG9*	F	GACGACAACTGGGGTCACTT
	R	TCGCAAATTATTCGGACACAG		R	GTGCTCGAGCTCTTGCTCTT
StATG8-1.2	F	CCAACAGGTGCAATCATGTC	*StATG10*	F	TTTGCATCCATGTGGGACTA
	R	CCCAATGTCAATACCATGAA		R	CTGACATAAGCCCCACCAAT
StATG8-2.1	F	CGGCTTCCTGTACATGACCT	*StATG11*	F	AACCTTCCTGCGAAAGACAA
	R	GCAGAGGGAAAATGAGAAAC		R	TGATCTCCAACTCCCTCACC
StATG8-2.2	F	TGAGGAACACAAGGATGAGG	*StATG13a*	F	ACATCATTTCCGTCGAGAGG
	R	CCATCTTAGCAAACGGGAAAT		R	CAGATATGTTGGTGGCGATG
StATG8-3.1	F	ATTCTCGCCGAACAGAGTTG	*StATG16*	F	CAATAGGCCTTTCGTTTTGC
	R	CCTCTCCAAAGGGTGTTCAA		R	TGCTTTAAGGTCCTGGTGCT
StATG8-3.2	F	GTGGTCCGAAAGAGGATCAA	*StATG101*	F	CCATCGTCTCCTCGATCAAT
	R	GAACAAATCAAAGTGGTAGC		R	GGGTGCTTCTCGACTCTGTC
StATG8-4	F	ACGGGATATAATCGCCAAAT			
	R	CTGCCACTCAGAATGTGGTT			
